# Ubiquitin in the immune system

**DOI:** 10.1002/embr.201338025

**Published:** 2013-12-27

**Authors:** Julia Zinngrebe, Antonella Montinaro, Nieves Peltzer, Henning Walczak

**Affiliations:** Centre for Cell Death, Cancer, and Inflammation (CCCI), UCL Cancer Institute, University College LondonLondon, UK

**Keywords:** autoimmunity, DUBs, E3 ligases, inflammation, LUBAC

## Abstract

Ubiquitination is a post-translational modification process that has been implicated in the regulation of innate and adaptive immune responses. There is increasing evidence that both ubiquitination and its reversal, deubiquitination, play crucial roles not only during the development of the immune system but also in the orchestration of an immune response by ensuring the proper functioning of the different cell types that constitute the immune system. Here, we provide an overview of the latest discoveries in this field and discuss how they impact our understanding of the ubiquitin system in host defence mechanisms as well as self-tolerance.

## Introduction

Our immune system's daily routine is a constant battle against attacks by invading potential pathogens. With a complex network of different cells, receptors and signalling pathways, the immune system ensures the elimination of pathogens, non-microbial foreign substances or damaged cells whilst also tolerating ‘self’ and commensal bacteria. The complexity, versatility and specificity of an immune response are accomplished not only by the variety of different cell types and receptors but also, and in particular, by post-translational modifications (PTMs) of proteins involved in immune signalling pathways. PTMs alter the properties of a protein by the addition of a modifying chemical group, or another protein, to one or more of its amino acid residues. The list of PTMs is long and to date more than 200 different PTMs have been identified; with phosphorylation, ubiquitination is likely to be the most extensively studied and best characterised of these 1 2. There is growing evidence of the importance of ubiquitination in the initiation, maintenance and termination of the immune system's response to many different stimuli.

An immune response requires tight regulation and control. Too little activation can cause immunodeficiency and recurrent infections, whereas too much activation can lead to autoimmunity, which is characterised by the recognition of self as non-self. An uncontrolled and sustained immune response can result in tissue damage and chronic inflammatory diseases.

## Ubiquitin and ubiquitination

Ubiquitination (also known as ubiquitylation or ubiquitinylation) is the energy-dependent, post-translational modification process in which the 8 kDa protein ubiquitin is covalently attached to one or more lysine residues of a substrate protein. The process of ubiquitination is a sequence of three events known as activation, conjugation and ligation, involving three different types of enzymes: the first step is ATP-dependent and carried out by a ubiquitin-activating enzyme, E1, and results in the formation of a thioester linkage between ubiquitin and the E1. The next step catalyses the transfer of ubiquitin from the E1 to the active-site cysteine of a ubiquitin-conjugating enzyme, E2. The third and final step of ubiquitination is executed by an E3 ubiquitin ligase resulting in the formation of an isopeptide bond between the C-terminal glycine of ubiquitin and a lysine residue of a target protein 3,4.

The substrate protein can also be ubiquitin itself, which will result in formation of a ubiquitin-linkage or di-ubiquitin. Ubiquitin has seven lysine residues, opening the possibility of forming seven different linkage types, namely K6, K11, K27, K29, K33, K48 and K63. In addition, a donor ubiquitin can also be attached to an acceptor ubiquitin via the amino terminal methionine (M1) resulting in the formation of M1- or linear linkages, making a total of eight different inter-ubiquitin linkage types 5.

The human genome encodes only two E1s, but at least 38 E2s and more than 600 E3s, making the process of ubiquitination very diverse and complex, but also very specific. It requires both linkage and substrate specificity, to ensure the attachment of the right ubiquitin linkage type to the intended substrate in the correct position. E2s are capable of mediating linkage specificity, either on their own, by the use of a cofactor or with the help of the respective substrate 6,7. In addition, some E3s possess intrinsic linkage specificity that is independent of the respective E2 8. Substrate specificity is ensured by the diverse E3s 9.

E3 ligases are divided into subfamilies according to their domain structure: HECT, RING or RBR ligases. HECT domains have been identified on the basis of sequence similarity to the C-terminal catalytic domain of the E3 ligase E6AP 10 whereas RING domains are characterised by a consensus sequence of cysteines and histidines that bind two zinc ions 9. HECT domains are always located at the C-terminus whereas RING domains can occur anywhere in an E3. The third subfamily, the RING-between-RING E3s, is defined by three domains that are in close proximity to each other: a classical RING domain, termed RING1, an in-between RING (IBR) domain, and a second RING domain, termed RING2. The IBR and RING2 domains are exclusively found in RBR E3s 11.

The domain organisation determines the mechanism of how ubiquitination occurs. In reactions involving HECT E3s the ubiquitin is transferred from the E2 to an active site cysteine of the E3 before being transferred to the substrate 12. E3s with RING domain structure are capable of bringing the ubiquitin that is bound to the E2 and the substrate into such close proximity that the transfer from the E2 to the substrate is a direct one. Recently, it has been shown that the mechanism for RBR ligases possesses features of HECT and RING E3s. RBR ligases first bind to the E2 that is complexed with ubiquitin via the RING1 domain representing the feature of classical RING E3s. However, RBR ligases require an additional step for substrate ubiquitination that involves an active site cysteine in the RING2 that catalyses the transfer of the ubiquitin from the E3 to the substrate and, thus, acts in a HECT-like manner. This RING-HECT hybrid mechanism has so far been shown for the RBR ligases HHARI, Parkin and HOIP [also known as ring finger protein (RNF) 31] 13,14,15.

Different ubiquitin linkages fulfill different physiological functions. The well-studied K48–linkage targets proteins for proteasomal degradation whereas K63-linkages are required for cell signalling events in DNA damage response or cytokine signalling 16,17. Until recently, the other linkage types were not studied in much detail and had therefore often been referred to as ‘atypical’ ubiquitin linkages. The functions so far attributed to atypical linkages are very diverse. For example, K6–linkages have also been implicated in the DNA damage response 18,19,20. K11-linkages were shown to mediate proteasomal degradation in mitosis and cell cycle regulation, but also to play a role in membrane trafficking and, most recently, in TNF signalling 21,22,23,24,25. K27–linkages were shown to be attached by the E3 ligase Parkin to mitochondrial proteins during mitochondrial damage response 26,27. In addition, K27–linkages are present on TIEG1, a transcription factor that is involved in the development of TGFβ-induced regulatory T cells (T_regs_) 28. Both, K29- and K33-linkages were implicated in the regulation of AMPK-related protein kinases 29. K33–linkages were further shown to be important for the regulation of T-cell receptor (TCR) responses 30. In 2006, M1- or linear ubiquitin linkages were shown to be catalyzed by a complex called LUBAC, consisting of two RBR ubiquitin ligases, HOIL-1 (also known as RanBP-type and C3HC4-type zinc finger-containing protein 1 (RBCK1)) and HOIP 5. The function of this type of linkage, however, was not revealed until 2009 when it became clear that linear ubiquitin chains are required for cell signalling events induced by TNF and Interleukin (IL)-1β 31,32. In addition, both HOIL-1 and HOIP were shown to form part of the native TNF-RSC I 31. In 2011, SHARPIN [also known as SHANK-interacting protein-like 1 (SIPL1)] was identified as the third component of LUBAC 25,33,34. Linear ubiquitin linkages have so far been implicated in the initiation and maintenance of gene activatory signalling upon activation with different stimuli, including TNF, IL-1β, CD40, muramyl dipeptide (MDP) and the endotoxin lipopolysaccharide (LPS), and in the prevention of TNF-induced cell death 25,31,32,33,34,35. Within these signalling pathways, NEMO, RIP1, RIP2 and IRAK1 have been identified as substrates for linear ubiquitination 25,36,37.

We are only beginning to understand the different signalling outcomes of differently linked chains, and additional research is required for the in-depth understanding and characterisation of these atypical ubiquitin linkages (Sidebar A).

**Sidebar A. In need of answers**

- What is the exact physiological role of the different ubiquitin linkage types?
- How do different linkage types orchestrate different signalling pathways and their outputs?
- How does a specific DUB recognise a specific linkage type?
- How is specific disassembly of ubiquitin linkages by a specific DUB achieved?
- How does deubiquitination of ubiquitinated substrates affect signalling output?
- What are the specific targets of DUBs within innate immune signalling pathways?
- Do different DUBs share common targets and, if so, is there a spatio-temporal regulation that makes them unique?
- How does ubiquitination/deubiqutination regulate inflammasome activation?
- Are there any additional E3 ligases that contribute to the maintenance of tolerance?
- How do the different E3 ligases cooperate to regulate the balance between immune activation and tolerance?

Proteins that contain so-called ubiquitin-binding domains (UBDs) non-covalently bind to ubiquitin and are therefore called ubiquitin-binding proteins (UBPs). More than twenty structurally different types of UBDs have been identified in over 150 different proteins. UBD-ubiquitin interactions enable conformational changes of UBPs, result in oligomerisation or complex formation of proteins, increase the affinity between the ubiquitinated substrate protein and the UBP, and critically determine signalling outputs, as UBDs specifically recognise certain linkage types. Our current knowledge on UBDs and UBPs has recently been summarised in excellent reviews 38,39,40. The specific recognition of certain ubiquitin linkage types by UBDs is essential for immune signalling pathways. NEMO, for example, contains the UBD UBAN, which specifically recognises linear chains, whereas TAB 2 and TAB 3 preferentially bind to K63-linkages via their UBD NZF 41,42,43. The *in-vivo* relevance of UBDs is emphasised by the fact that mutations that affect the UBAN domain of NEMO, and thereby binding of NEMO to polyubiquitin chains, result in defective NF-κB activation and disease development (reviewed in 44).

**Figure 1:**
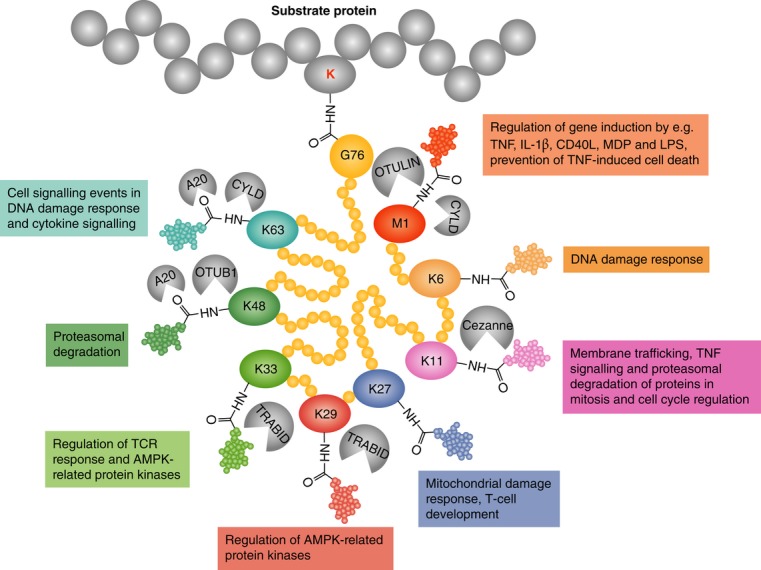
Different ubiquitin linkage types and their role in immune signalling Ubiquitin, a small protein of 76 amino acids, can be attached to a substrate protein or to a ubiquitin molecule that is already attached to a substrate, with the latter resulting in an inter-ubiquitin linkage. Attachment to substrates will typically occur through an isopeptide bond between the ε-amino group of a lysine residue (K) within the substrate protein and the C-terminal carboxyl group of glycine 76 (G76) of ubiquitin. Inter-ubiquitin linkages are usually between the ε-amino group of one of the seven internal lysine residues (K6, K11, K27, K29, K33, K48 and K63) of the substrate-associated acceptor ubiquitin and the carboxyl group of G76 of the incoming ubiquitin*. Another type of inter-ubiquitin linkage can be formed between the α-amino group of the N-terminal methionine 1 (M1) of the substrate-associated ubiquitin and the carboxyl group of G76 of the incoming ubiquitin. The resulting linkage type is called M1- or linear linkage. Different inter-ubiquitin linkage types fulfil different functions in immune signalling. The functions currently attributed to the different linkage types are summarised in the respective boxes. Removal of ubiquitin is carried out by DUBs, which have recently been shown to specifically cleave certain linkage types, for example CYLD cleaves K63- and M1–linkages.

The termination of an immune response is as important as its initiation and propagation. Termination is achieved by the removal of ubiquitin chains that mediate cell signalling events, such as K63-linkages, and subsequent attachment of chains that mark a specific protein for degradation. Removal of ubiquitin is carried out by so-called de-ubiquitinating enzymes (DUBs). The human genome encodes approximately 80 different DUBs. DUBs were identified to have specificity for certain linkage types 45,46: K11-linkages are cleaved by Cezanne 1 (also known as OTU domain-containing protein 7B), TRABID (also known as ZRANB1) was shown to cleave K29- and K33-linkages 47,48, OTUB1 cleaves K48-linkages, and CYLD (encoded by the cylindromatosis gene) cleaves K63- and M1-linkages 43,49. A20 was identified as a negative feedback regulator of NF-κB by cleaving K63–linkages on NF-κB signalling molecules, for example RIP1 50,51. The OTU domain of A20, however, was shown to preferentially cleave K48-linkages *in vitro*, suggesting that additional factors are required to modulate A20's linkage specificity 43,52. A20 was recently shown to restrict RIP1 ubiquitination by cleaving both, K48- and K63-linked chains 53. Interestingly, A20 contains seven zinc fingers (ZF), of which ZF4 was shown to have intrinsic E3-ligase activity that is required for binding to and K48-linked ubiquitination of RIP1 51,54. A DUB that exclusively cleaves linear linkages was also recently discovered; due to this activity, it was named OTULIN (also known as Gumby or Fam105B) 55,56. Also DUBs harbour UBDs and the recognition and binding of a certain UBD to a specific ubiquitin linkage is thought to be essential for specific chain hydrolysis 45. However, how a particular DUB recognises a particular linkage type and how cleavage by a given DUB regulates specific signalling events requires further investigation (Sidebar A). The different linkage types, their known biological functions and respective DUBs identified so far are summarised in Fig1.

### The immune response

The first lines of defence are the body's physical barriers, such as the skin or the intestinal and respiratory tracts. These physical barriers possess multiple non-specific defence strategies that comprise mucus production, decontamination by acid or special enzymes, and clearance of airways by cilia 57. When these physical barriers are pervaded by pathogens, the second line of defence, the innate immune system, is activated. The innate immune system detects microbial products, the so–called pathogen-associated molecular patterns (PAMPs), via the family of pattern-recognition receptors (PRRs) on both immune and non-immune cells 58,59. The PRR family consists of different subfamilies, namely the Toll-like receptors (TLRs), the RIG-I-like receptors (RLRs) and the NOD-like receptors (NLRs) 60,61. These receptors will not only be activated by microbial products, but many of them also sense damage signals, the so-called damage-associated molecular patterns (DAMPs) 62. These alarmins are released by damaged, injured or stressed cells and are usually cytosolic or nuclear proteins. Activation of PRRs will result in the activation of mitogen-activated protein kinase (MAPK) and NF-κB signalling, and subsequent induction of inflammatory cytokines and chemokines —such as TNF, IL-1β, etc. These cytokines and chemokines will lead to an inflammatory response and to the recruitment of immune cells to the site of infection or tissue damage 63. The innate immune system provides an immediate but non-specific response. A long-lasting and very specific immune response is achieved by the activation of the adaptive immune system, the third and final line of defence. Antigen-presenting cells (APCs), which belong to the innate immune system, engulf pathogens and provide a link between the innate and the adaptive immune system, the B and T cells 64,65,66. T cells strongly depend on presentation of antigens by APCs for their activation. Cytotoxic T cells are capable of directly attacking and killing infected cells. Helper T cells, as the name implies, help other immune cells to differentiate and fulfill their function. The activation of B cells results in clonal expansion of a pathogen-specific B cell and generation of antibody-producing plasma cells or memory B cells. Memory B cells ensure that a second contact with the same antigen will lead to a faster and stronger immune response and elimination of the invader 57.

The sequence of events of an immune response requires tight control, and ubiquitination and its reversal, deubiquitination, seem to be crucial for the induction of an adequate but confined immune response.

Recent advances in the understanding of ubiquitination events in innate immune signalling pathways will be the focus of this review. However, adaptive immune signalling pathways have also been shown to require ubiquitination for proper signal transduction 67, and ubiquitination seems to have an essential role in directing the development of different adaptive immune cell lineages at the transcriptional level 68.

## Ubiquitin in innate immune signalling

### The PRR family

#### TLR signalling

The TLRs are a family of membrane-bound receptors responsible for sensing a broad array of pathogens—including bacteria, fungi, viruses and protists—both outside the cell and intracellularly in endosomes and lysosomes 60,69,70. Indeed, TLRs are divided into two groups according to their subcellular localisation: endosomal TLRs (TLR 3, 7, 8 and 9) engage luminal PAMPs from endocytosed pathogens, and plasma-membrane-expressed TLRs (TLR1, 2, 4, 5, 6 and 10) sense extracellular PAMPs. TLR engagement leads to recruitment of a series of proteins resulting in a signalling cascade that culminates in induction of pro-inflammatory cytokines and/or type-I interferons (IFNs). Alternatively, TLR signalling can lead either directly or indirectly (through the production of TNF, described later in this review) to the induction of cell death through RIP1, RIP3, caspase-8, FADD and cFLIP 71,72,73. Our knowledge of the role of ubiquitination in TLR-induced cell death is still rather limited. Therefore, this review will focus on the role of ubiquitination in gene-regulatory signalling downstream of TLR3 and TLR4 by the two different adaptor proteins that can be engaged, namely MyD88 or TRIF 74, which account for different signalling outputs.

**Figure 2:**
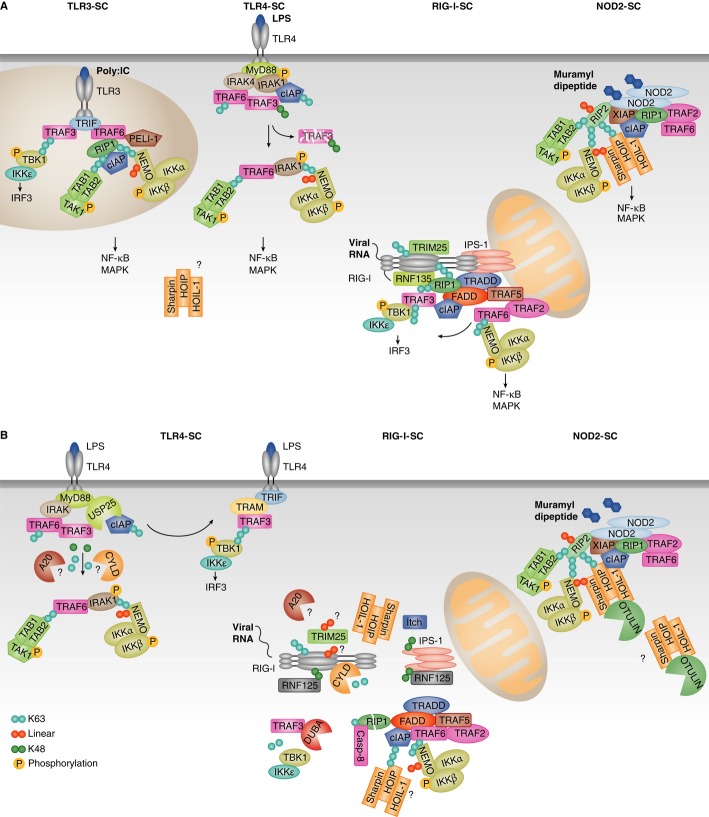
Ubiquitination and deubiquitination are involved in multiple PRR signalling complexes (A) Multiple E3 ligases have been shown to be required for maximal gene activation in TLR3, TLR4, RIG-I and NOD2 signalling. Stimulation of TLR3 in the endosome results in recruitment of TRIF and the E3 ligases TRAF3 and TRAF6. TRAF3 enables further recruitment of the TBK1/IKKε kinase complex resulting in IRF3 activation and subsequent IFN production. TRAF6 mediates recruitment of PELI-1 via K63-linkages. PELI-1 in turn attaches K63-polyUb chains to RIP1. In addition, TRAF6 is required for recruitment of TAB/TAK and IKK kinase complexes, resulting in NF-κB activation. The E3 ligases cIAP1 and cIAP2 are also recruited to the complex. Similarly, stimulation of TLR4 by LPS results in receptor complex formation on the plasma membrane. This complex consists of MyD88, IRAK1/4, TRAF3/6 and cIAP1/2. cIAP1/2-mediated K48-poly-ubiquitin linkages on TRAF3 result in its proteasomal degradation and the formation of a secondary cytoplasmic complex. LUBAC has been suggested to regulate TLR signalling. RIG-I activation upon virus recognition is driven by TRIM25- and RNF135-mediated K63-poly-ubiquitination and oligomerisation of RIG-I. Activated RIG-I binds to and activates the adaptor protein IPS-1 in the mitochondrial membrane, which induces the recruitment of TRADD, FADD and RIP1 and subsequent recruitment of TRAF2/3/5/6 and cIAP1/2, resulting in TBK1/IKKε-mediated IRF3 and TRAF2/5/6-mediated NF-κB activation. NOD2 is an intracellular receptor that detects bacterially derived muramyl dipeptide (MDP), leading to the formation of a complex consisting of RIP1, TRAF2/6, cIAP1/2 and XIAP, and RIP2. Ubiquitination of RIP2 is a key event in NOD2 signalling, which results in the recruitment of LUBAC. (B) LUBAC, although a positive modulator in most SCs was shown to negatively regulate RIG-I-signalling by preventing TRIM25/RIG-I interaction or by linear ubiquitination of NEMO and prevention of TRAF3 recruitment. RIP1 poly-ubiquitination has both a positive and negative regulation on RIG-I-signalling, as it is needed for its recruitment as well as cleavage by caspase-8 at the complex. RNF125 catalyzes the formation of K48-linked polyUb chains in both RIG-I and IPS-1, thereby inducing their proteasomal degradation. Several DUBs regulate these signalling complexes. USP25 binding to MyD88 results in removal of K48-linkages from, and consequently stabilisation of, TRAF3. TRAF3 is then recruited to a TLR4/TRIF/TRAM complex leading to IRF3 activation. A20 and CYLD have been shown to modulate these receptors although the exact mechanisms are still poorly understood. In NOD-2 signalling, A20 is known to deubiquitinate RIP2. OTULIN has been described to selectively hydrolyse M1-linkages, thereby preventing NOD2 signalling under both basal and stimulated conditions.

MyD88-dependent signalling, engaged by TLR4 but not TLR3, induces the recruitment of IRAK proteins that subsequently interact with TRAF6, TRAF3, cIAP1 and cIAP2 74,75 (Fig2A). Within this TLR4 signalling complex (TLR4-SC) TRAF6 is activated and conjugates K63-linkages to itself and to cIAP1/2, that serve as a scaffold for the recruitment of two kinase complexes, namely TAB-TAK and IKK-NEMO, leading to the activation of NF-κB and MAPK 42,76. Importantly, the activation of the TAB-TAK complex requires detachment of the TLR4-SC from the plasma membrane and translocation to the cytosol. This process requires cIAP1/2-mediated K48-ubiquitination of TRAF3 leading to its proteasomal degradation, thereby allowing the residual complex to migrate to the cytosol 77, resulting in activation of the TAB-TAK complex and transcription of inflammatory cytokines.

Upon stimulation, TLR3 and TLR4 recruit the adaptor protein TRIF although TLR4, but not TLR3, requires the additional recruitment of another adaptor protein, TRIF–related adaptor molecule (TRAM) to signal through TRIF (Fig2A and B). Depending on the ubiquitin ligases recruited to the complex, endosomal TRIF-dependent signalling results in activation of NF-κB and/or type-I IFNs. In the TLR3-SC RIP1 is recruited to a complex formed by TRIF, TRAF6 and the E3 ligase PELI-1 which belongs to the pellino family. PELI-1 subsequently attaches K63-linked polyubiquitin linkages to RIP1 78, resulting in further recruitment of IKK-NEMO and TAB-TAK complexes to induce NF-κB-mediated transcription of pro-inflammatory cytokines. Alternatively, and contrary to MyD88-dependent signalling in which TRAF3 acts as a negative regulator, TRAF3 undergoes K63-linked polyubiquitination (polyUb) and activates the TBK1/IKKε complex, leading to IRF3 phosphorylation and subsequent type-I IFN production 79,80.

Other ubiquitin ligases have been shown to play an important role in TLR signalling although the exact mechanism is less well characterised. cIAPs are known to be involved in TLR3 signalling and although their role in NF-κB activation has not been described, they are known to be recruited to the TLR3-SC and to regulate cell death induction via RIP1 polyubiquitination 71,72. Additionally, an increasing amount of evidence suggests that LUBAC is involved in TLR-mediated signalling. Macrophages derived from mice that are devoid of one of the LUBAC components, SHARPIN (also known as *cpdm* mice; described later in this review)*,* are unable to induce phosphorylation of IκBα upon engagement of TLR4 by LPS, a well-known TLR4 ligand 34. Similarly, bone marrow-derived dendritic cells (BMDCs) from *cpdm* mice failed to activate NF-κB upon stimulation with LPS or poly(I:C), a TLR3 ligand 81. Furthermore, a recent study by Sasaki and colleagues showed that B cells derived from mice lacking the E3 ligase activity of HOIP have impaired NF-κB and ERK activation upon LPS stimulation 82. Although LUBAC clearly plays a role in TLR-mediated signalling —mostly by activating TAB-TAK and IKK-NEMO complexes— the mechanism by which LUBAC regulates TLR-mediated signalling is not well understood and therefore currently intensely studied.

To avoid unwanted exacerbated immune responses, DUBs are as important in this process as the ubiquitin ligases themselves. Recently, ubiquitin-specific protease (USP)-25 has been described to play an essential role in shutting off TLR4-mediated induction of MAPK signalling 83. USP25 interacts with MyD88, but not with TRIF, restricting the production of pro-inflammatory cytokines whilst promoting IFN production via stabilisation of TRAF3 in LPS-stimulated DCs, macrophages and MEFs. Mechanistically, USP25 acts as a direct TRAF3 DUB, and thereby counteracts cIAP1/2's inhibitory effect on TRAF3, preventing the dissociation of the complex from the membrane and restricting the pro-inflammatory response 83. Concomitantly, the stabilisation of TRAF3 by USP25 promotes the TRIF-dependent production of type-I IFN (Fig2B). This dual function of USP25 may maintain the balance between TLR-triggered production of pro-inflammatory cytokines and type-I IFNs. The DUB USP7 has recently been proposed to be an important NF-κB regulator in response to diverse TLR stimuli, including LPS and poly(I:C). USP7 stabilizes NF-κB at target gene promoters by deubiquitinating it and thereby preventing its proteasomal degradation. Ubiquitination and deubiquitination by E3 ligases and USP7, respectively, have been proposed to determine the strength and duration of the transcriptional outcome of NF-κB-activated genes 84.

Another important DUB involved in TLR-mediated response is A20. A20-deficient mice develop spontaneous inflammation and die shortly after birth 50. This is rescued by co-ablation of MyD88, but not of TNF or TNFR1, indicating that signalling by MyD88-recruiting TLRs is the main mediator of A20-deficiency-dependent inflammation 85,86. A20 is part of a multi-protein complex that includes TAX1BP1, the E3 ubiquitin ligases Itch, RNF11 and ABIN1/2/3 87,88. Further supporting the importance of A20 in TLR mediated signalling, TAX1BP1-deficient macrophages and fibroblasts enhance K63-linked ubiquitination of TRAF6 upon LPS stimulation, and it was suggested that it functions as an adapter molecule that links A20 to its substrate, in this context, TRAF6 89. Finally, the DUB CYLD might modulate TLR signalling, as demonstrated in CYLD-deficient macrophages, which were found to be hyper-responsive to LPS stimulation. However, the specific CYLD target modulating TLR3 and TLR4 responses remains to be determined 90 (Sidebar A).

#### RLR signalling

RLRs are cytosolic PRRs involved in sensing actively replicating double-stranded RNA (dsRNA) viruses. There are three RLRs, RIG-I, MDA5 and LGP2 67. Upon sensing viral particles, RIG-I becomes K63-ubiquitinated by two E3 ligases, TRIM25 and RNF135, but also by unanchored (free) K63-polyUb chains, leading to RIG-I oligomerisation, activation and binding to the adaptor protein IPS-1 (also known as MAVS, VISA and Cardif) at the mitochondrial outer membrane 69,91 (Fig2A). Activated IPS-1 recruits TRADD, which in turn recruits multiple components to trigger the antiviral response 92. Recruitment of the E3 ligase TRAF3 leads to further recruitment of the TBK1 complex, resulting in the activation of IRF3 and subsequent IFN production 93. On the other hand, recruitment of TRAF6, together with the adaptors TRAF2 and TRAF5, is involved in NF-κB activation, although a recent study reported that this E3-ligase/adaptor complex can also mediate IRF3 activation, and that TRAF3 might have a redundant function 92. TRADD also recruits caspase-8, RIP1 and FADD, which are required for proper activation of IRF3 and NF-κB 93,94 (Fig2A). Ubiquitination of RIP1 has been suggested to have a dual regulatory function on RIG-I-mediated signalling, being involved in both the maximal activation of antiviral signalling and its subsequent proper termination. Although RIP1 ubiquitination seems to positively regulate RIG-I signalling, it is also a prerequisite for its cleavage by caspase-8 in the RIG-I-signalling complex (SC), resulting in a negative regulation of RIG-I-mediated type-I IFN production 95 (Fig2A and B). The ubiquitin linkage types on RIP1 that are involved in this dual function are unknown, but both cIAP1/2 and LUBAC may play a role in this tight regulation (see below).

E3 ligases such as cIAP1/2, RNF125 and LUBAC are also involved in RIG-I-mediated signalling 96. cIAP1/2 attaches K63-polyubiquitin linkages to TRAF3, TRAF6 97 and possibly other components, thereby positively affecting activation of IRF3 and NF-κB. RNF125, on the contrary, has an inhibitory effect on RIG-I-SC, as it attaches K48-linked polyUb chains to both RIG-I and IPS-1, targeting them for proteasomal degradation 98 (Fig B). Surprisingly, LUBAC was also reported to have a negative regulatory function in RIG-I-mediated antiviral responses through supposedly two different mechanisms (Fig2B). On the one hand, Inn and colleagues showed that LUBAC prevents RIG-I-SC assembly through the ubiquitin-mediated degradation of TRIM25 and/or disruption of the TRIM25-RIG-I interaction, both scenarios resulting in inhibition of TRIM25-mediated K63-ubiquitination of RIG-I, thereby preventing RIG-I's interaction with mitochondrial IPS-1. Accordingly, Sendai virus (SeV) infection induced linear- and K48-polyubiquitin linkages on TRIM25, and infection of HOIP knockdown cells led to increased IFN production 99. On the other hand, Belgnaoui and colleagues reported that, LUBAC-mediated linear ubiquitination of NEMO sequesters TRAF3 and prevents it from binding the RIG-I/IPS-1 complex. In line with this, vesicular stomatitis virus (VSV)-infected *cpdm*-derived MEFs produced less virus and had an increased antiviral response due to higher IFN production 100. It therefore seems that, in the context of RIG-I-mediated antiviral responses, linear ubiquitination has an opposing role to that determined for other signalling pathways such as those triggered by TNF 25,31, IL-1 37, TLR 82 and NOD2 35,36. The precise role of linear ubiquitination in RIG-I signalling requires further investigation to be fully understood (Sidebar A).

Several DUBs were shown to have an inhibitory effect on the RIG-I signalling pathway (Fig2B), as for example knockdown of CYLD enhanced type-I IFN production in response to SeV infection whereas ectopic CYLD-expression inhibited it 101. Furthermore fibroblasts and BMDCs from CYLD-deficient mice were shown to have constitutive activation of TBK1. Mechanistically, CYLD was shown to interact and deubiquitinate RIG-I, therefore inhibiting its signalling function 102. Another DUB suggested to play a role in RIG-I signalling is DUBA. Overexpression of DUBA results in deubiquitination of TRAF3 thereby disrupting the TRAF3-TBK1 interaction 103 (Fig2B). Given that TRAF3 may be dispensable for RIG-I signalling 92, it cannot be excluded, however, that DUBA also targets other E3 ligases like TRAF2, 5 and 6. In addition, A20 has been shown to counteract RIG-I-mediated transactivation of IRF3 and NF-κB pathways but direct interaction of A20 with RIG-I or IPS-1 has not been reported 87,104. Furthermore, the E3 ligase Itch, which forms part of the A20 complex, has been shown to attach K48-linked polyUb chains to IPS-1, leading to its proteasomal degradation. However, similarly to A20, it has not been shown to interact directly with IPS-1 105.

#### NLR signalling

The NLR family are cytoplasmic sensors that detect bacterial infections. Two of the best studied NLRs, NOD1 and NOD2, detect the presence of peptidoglycans derived from bacterial cell walls. Interestingly, mutations in NOD2 have been linked to inflammatory bowel disease.

Stimulation of NOD1 or NOD2 triggers the formation of a signalling complex (NOD-SC) resulting in the activation of cytokines, chemokines and antimicrobial peptides 69. The NOD-SC has been reported to contain RIP2 and cIAP1/2, and the cIAPs conjugate K63-ubiquitin linkages to RIP2 35,106 (Fig2A). In addition, TRAF2, 5 and 6 are recruited to the NOD-SC, where they function as adaptors and are important for the crosstalk between several NLRs, TLRs and RLRs 107. Another known IAP, XIAP is also recruited to the NOD-SC, where it directly binds to RIP2 and plays a major role in its ubiquitination 35,108. Furthermore, XIAP is responsible for the recruitment of LUBAC 35, which conjugates linear ubiquitin linkages to RIP2 36 and possibly also to NEMO. Together, K63- and M1-linkages are essential for the efficient recruitment and activation of TAB-TAK and IKK-NEMO complexes, ultimately leading to activation of NF-κB and MAPK signalling and subsequent induction of inflammatory mediators in NOD signalling 109.

The importance of XIAP in the innate immune response to intracellular bacteria has been shown in several infection models. Upon C. pneumoniae infection, XIAP-deficient mice suffer from increased pulmonary infectivity and are unable to clear bacteria as a consequence of reduced NF-κB activation 110. Similarly, XIAP-deficient mice succumbed to an intraperitoneal injection of Listeria monocytogenes due to increased bacterial burden 111. Co-activation of NOD2 and TLR4 was shown to lead to synergistic production of cytokines required to mount an efficient immune response against bacteria 112. Fatal hepatitis, induced by injection of LPS together with the liver-specific transcriptional inhibitor GalN and the NOD2 ligand MDP, was accelerated in XIAP-deficient mice. This effect was NOD2-signalling-dependent, as livers of XIAP-deficient mice showed only mild damage when treated with LPS and GalN alone 35. In line with this, cytokine levels were reduced in serum and in bone marrow-derived macrophages (BMDMs) derived from XIAP-deficient mice upon MDP treatment. Importantly, this was independent of the role of XIAP as an inhibitor of apoptosis since treatment with MDP and/or LPS neither induced cell death in wild-type nor in XIAP-deficient macrophages 35. Together, these results underscore the requirement of XIAP's E3 ligase activity in NOD signalling.

Importantly, recent work performed in cells obtained from patients with the X-linked lymphoproliferative syndrome 2 (XLP2) gave important insight on NOD signalling in humans. This disease is caused by mutations in the *Xiap* gene, either in the part that encodes the RING domain or the one that encodes the BIR2 IBM-binding pocket. XLP2-causing mutations in the RING domain abrogate XIAP's ubiquitin ligase activity whereas mutations in the BIR2 domain impair XIAP binding to RIP2 and both defects lead to an impaired NOD2-mediated NF-κB activation 113. The SMAC mimetic Compound A displaced XIAP-RIP2 binding, thereby antagonising RIP2 ubiquitination and recruitment of LUBAC. Importantly, this was independent of cIAP1/2, as it occurs in the XLP2 patients 113. This suggests that NOD1/2 signalling might be regulated at the level of XIAP recruitment to the NOD-SC by BIR2-IBM binding proteins and other factors that interfere with its binding to RIP2, rather than by cIAP1/2. In line with this work, the inositol phosphatase SHIP-1 was reported to negatively regulate NOD2 signalling by interfering with XIAP-RIP2 binding 114.

The DUB A20, which directly deubiquitinates RIP2, is a negative regulator of NOD2-SC. Consistently, exacerbated responses to MDP stimulation, with increased RIP2 ubiquitination and NF-κB activation, were observed both *in vitro* and *in vivo* in the absence of A20 115. Similarly, the recently identified DUB OTULIN 36,55,56 negatively regulates NOD2-mediated signalling by preventing LUBAC autoubiquitination under basal conditions, as well as restricting the accumulation of linear ubiquitin chains on RIP2 and LUBAC upon stimulation (Fig2B). OTULIN depletion in cells resulted in increased MDP-induced transcription of NF-κB target genes, and its overexpression inhibited LUBAC- and, to a lesser extent, XIAP-mediated NF-κB activation 36.

An emerging role of ubiquitination has also been described for another important NLR, the NLRP3, which is involved in the assembly of the inflammasome —a platform containing caspase-1 that is involved IL-1β activation 116. cIAP1/2 have both been implicated in inflammasome regulation, although some controversy in their function makes it currently difficult to fully understand their exact influence and mechanism of action. Labbé and colleagues reported that cIAP1/2, together with TRAF2, directly interact with a caspase-1-containing complex and mediate its K63-polyubiquitination, thereby positively regulating inflammasome activation. Consistently, cIAP1/2 inhibition in THP-1 cells, a monocytic cell line, markedly inhibited caspase-1 activation and IL-1β processing 117. Vince and colleagues, on the contrary, demonstrated that cIAP1/2 and XIAP have an inhibitory effect on the NLRP3-inflammasome. The authors showed that either treatment with the SMAC mimetic Compound A or genetic ablation of cIAP1/2 and XIAP induced rapid generation of the NLRP3-caspase-1 inflammasome, leading to increased IL-1β maturation and secretion. They further showed that, in the absence of IAPs, activation of IL-1β secretion can occur in a caspase-1-independent manner, suggesting the existence of other IL-1β-activating platforms, possibly relying on caspase-8, in which IAP E3 ligase activity exerts an inhibitory effect 118. Although the exact roles of cIAP1 and cIAP2 remain to be established, it is clear that they have a crucial role in inflammasome activation. Py and colleagues recently identified the DUB BRCC3 as a positive regulator of the inflammasome by directly deubiquitinating NLRP3. BRCC3 knockdown leads to an increase in NLRP3 ubiquitination, and the DUB activity of BRCC3 is required for caspase-1 activation and IL-1β processing, but not for LPS-induced transcription of pro-IL-1β or of NLRP3 itself 119.

Taken together, these recent studies provide new insight on the importance of ubiquitination in NLRP3-inflammasome-dependent and -independent IL-1β activation. Investigation of the role of other E3 ligases and DUBs in inflammasome activation will further contribute to a more comprehensive understanding of this process (Sidebar A).

### The TNF superfamily

The TNF superfamily consists of 19 members, which share sequence homology with its founding member TNF. These ligands interact with 29 different receptors, resulting in a plethora of physiological outcomes, including inflammation, proliferation and cell death 120. Ubiquitination has been identified to regulate many, if not all, of the signalling pathways used by these ligands 25,31,82,121. TNF signalling has always been the exemplar and best-understood signalling pathway of the TNF superfamily. However, the more we find out about the complexity and accuracy of the sequence of events in TNF signalling, the more we realise what remains to be discovered. Recent advances in the field, which further unravelled the importance of ubiquitination for the sequence of events induced by ligation of TNFR1 with TNF will be discussed below. However, more work will be required to decipher the ubiquitin code of TNF signalling (Sidebar A).

#### TNF signalling

TNF stimulation results in two opposing signalling outcomes. On the one hand, TNF is a potent inducer of pro-inflammatory and cell-death-preventing signalling cascades via TNF-RSC I. On the other hand, TNF stimulation results in elimination of infected or damaged cells via the induction of cell death by a cytosolic complex that consecutively forms out of TNF-RSC I, termed complex II 122. Although the induction of gene-activatory signalling is the major role of TNF, its cell-death-inducing capacity was the function that gave this cytokine its name in 1975 123. Carswell and colleagues identified the production of TNF by endotoxins to cause endotoxin–mediated tumour necrosis, a phenomenon that had been known since 1890 and was first described by William Coley 124.

The sequential transition from membrane-bound TNF-RSC I into cytosolic complex II implies that signalling by TNF always results in gene activation prior to possibly inducing cell death. Both signalling outputs of TNF can be useful and essential for the host response against invading pathogens. However, apart from being beneficial and important for the induction of an adequate immune response, inordinate TNF stimulation can also result in fulminant pathological outcomes; for example, excessive TNF production was identified as mediator of lethal septic shock 125,126. Thus, TNF signalling is tightly controlled, and ubiquitination and deubiquitination are crucial components in this regulation.

**Figure 3:**
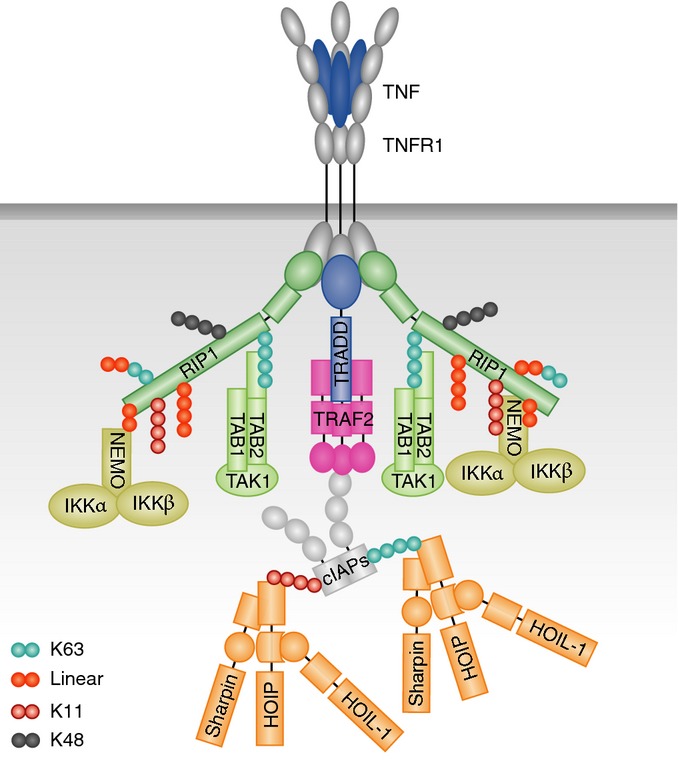
Different linkage types in the TNFR1-signalling complex orchestrate the TNF signalling output Crosslinking of TNFR1 by TNF results in trimerisation of TNFR1 and recruitment of TRADD and RIP1 via their DDs. This results in subsequent recruitment of TRAF2 and in turn cIAP1/2. cIAP1/2 place different lysine-linked ubiquitin chains (K11, K48 and K63) on various components of the TNF-RSC. This activity is required for recruitment of LUBAC, which subsequently places linear ubiquitin linkages (M1) on RIP1, NEMO and possibly other components. Both cIAP1/2 and LUBAC may also create K63-M1-hybrid chains on RIP1. The different ubiquitin linkages placed by cIAPs and LUBAC enable and orchestrate the physiologically required gene-activatory capacity of this complex by ensuring exact positioning of both the IKK and TAK/TAB complexes. Different ubiquitin linkages are indicated in different colours. The exact positions of these chains have not yet been identified, and the precise lengths and linkage sequences of these chains remain to be established.

Binding of TNF to TNFR1 leads to receptor trimerisation and recruitment of both, TRADD and RIP1 via their death-domains (DDs) 127,128,129. TRADD further recruits TRAF2, or alternatively TRAF5, which in turn leads to recruitment of the E3 ligases cIAP1/2 128,130,131,132,133. The recruitment of both TRAF2/5 and cIAP1/2 is required for K63-linked ubiquitination of RIP1, downstream recruitment of TAB/TAK and IKK-NEMO complexes, and subsequent activation of NF-κB and MAPK signalling 76,134 (Fig3). It was long thought that K48- and K63-linkages were the only ubiquitin linkages required for TNF signalling. This view was challenged by three discoveries. First, Xu *et al* (2009) 135 demonstrated that K63-linkages are dispensable for TNF-induced NF-κB activation, suggesting that either other linkage types are capable of replacing K63-linkages or that K63-linkages are not required for NF-κB activation. Second, it was discovered that linear ubiquitination, mediated by LUBAC, is essential for full TNF-mediated NF-κB activation 31,32. Importantly, the tripartite complex LUBAC consisting of SHARPIN, HOIL-1 and HOIP was not only identified as a new component of the native TNF–RSC I, but also shown to mediate linear ubiquitination of RIP1 and NEMO 25,31. Third, apart from linear linkages, K11-, K48- and K63-linkages were found to be simultaneously present on RIP1 in TNF-RSC I, proposing that additional linkage types are required to orchestrate and modulate TNF's signalling output 24,25 (Fig3).

LUBAC is recruited in a TRADD-, TRAF2/5- and cIAP1/2-dependent manner, and recruitment of LUBAC is critically dependent on the E3-ligase activity of cIAP1/2 31. This suggests that LUBAC most likely binds to cIAP1/2-mediated linkages in TNF-RSC I. LUBAC was shown to be a decisive regulator of TNF signalling since its presence stabilises TNF-RSC I resulting in maintained NF-κB and MAPK signalling whereas its absence leads to enhanced TNF-mediated cell death via complex II. Importantly, stabilisation of TNF-RSC I and inhibition of transition into complex II is not mediated by the mere presence of LUBAC components in the complex but requires LUBAC's E3 ligase activity 31. Apart from being a substrate of linear ubiquitination, NEMO was shown to bind linear linkages with 100-fold higher affinity than K63-linkages through its UBAN domain 41,136,137, suggesting that LUBAC, once recruited, orchestrates the sequence of events by enabling recruitment of NEMO via linear linkages on TNF–RSC I components, like RIP1 and possibly others. More research will be required to unravel the distinct roles and functions of different ubiquitin linkages on the various components involved in TNF signalling.

TNF signalling is controlled by different DUBs. Negative feedback regulation of TNF-induced NF-κB activation is ensured by the NF-κB-dependent upregulation of expression of the DUBs A20 and CYLD 138. A20 mediates the cleavage of K63-linked chains on RIP1, and subsequently marks RIP1 —through the attachment of K48-linked chains— for proteasomal degradation 51. A20 thereby terminates TNF-induced NF-κB activation and at the same time prevents TNF-induced cell death. CYLD, a DUB that can cleave both K63- and linear linkages, was shown to negatively regulate NF-κB activation by removing K63–linkages from RIP1 139,140,141,142. But in contrast to A20, CYLD is required for TNF-induced cell death, and cleavage of CYLD by caspase-8 prevents programmed necrosis, termed necroptosis 140,142,143. Whether CYLD also cleaves linear linkages in TNF-RSC I, and how this impacts the TNF signalling output, is currently unclear. The recently identified linear-chain-specific DUB OTULIN has been implicated in the regulation of TNF–induced NF-κB activation and cell death 55. However, whether OTULIN forms part of the native TNF–RSC I remains to be determined.

Deubiquitination of RIP1 seems to be a prerequisite for complex II formation, as RIP1 ubiquitination was shown to protect from cell death 143,144. Whether complex II induces apoptosis or necroptosis depends on the activity of individual components of the complex. Apoptosis is induced by complex IIA, which consists of TRADD, FADD, caspase-8, RIP1 and RIP3. Caspase-8 constitutively inactivates RIP1 and RIP3, thereby allowing apoptosis induction. Absence of caspase-8 or caspase-8 activity results in phosphorylation of RIP1 and RIP3 and subsequent RIP1/RIP3-dependent necroptosis induction via complex IIB, also called the necrosome 145,146. Little is known about specific ubiquitination and deubiquitination events on components of complex II, but they likely also play a crucial role in determining the output of this signalling complex.

## Ubiquitin in autoimmunity and autoinflammation

What is commonly referred to as autoimmunity was first cited at the turn of the 20th century by Paul Ehrlich, who used the phrase ‘horror autotoxicus’ to define the processes in which the immune system attacks self. Five decades later, Burnet and colleagues provided a theoretical basis for autoreactivity by demonstrating the presence of autoantibodies 147. This concept is currently viewed as a defect of the immune system concerning either B or T cells involved in adaptive immune activation, which can lead to autoimmune diseases characterised by tissue damage.

There are two main processes that play a key role in the establishment of the immune system's tolerance to self-antigens: central and peripheral tolerance. The induction of central tolerance takes place in the thymus, where medullary thymic epithelia cells (mTECs) and medullary DCs present a broad array of self-peptides in association with specialised proteins known as the major histocompatibility complexes (MHC) on their surface to developing T cells. The T cells whose TCR recognises a self-peptide in the context of an MHC molecule with an affinity that is above a certain threshold are eliminated. This process is called negative selection and aims to prevent host tissue damage by autoreactive T cells.

Peripheral tolerance is an additional strategy to ensure that activation and function of autoreactive T cells that might have escaped negative selection in the thymus are kept under control. Several mechanisms contribute to peripheral tolerance: (i) hypo-responsiveness to antigens expressed at low levels 148, (ii) deletion of activated T cells through antigen-induced cell death 149, (iii) T cell intrinsic functional inactivation (anergy) 150, and (iv) active suppression by the action of regulatory T cells (*T*_regs_) 151,152.

**Figure 4:**
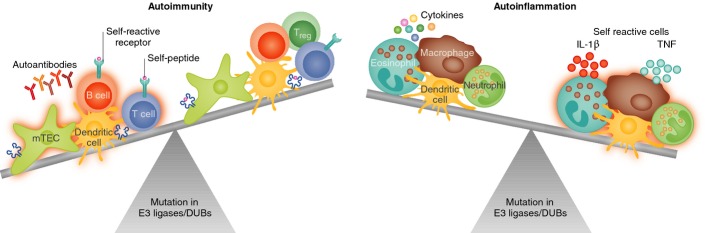
Ubiquitin and immune system modulation Cells of both the innate and adaptive arms of the immune system are crucial for immune reactivity and self-tolerance. Defects in the expression of E3 ligases or DUBs can lead to deregulation of these processes which can resulting in autoimmunity or autoinflammation. Autoimmune disorders are characterised by disturbed function of mTEC and DCs (impairment in their presentation of self-peptides to T cells; highlighted in red) or by perturbed peripheral tolerance, which will lead to the development of self-reactive T and/or B cells (highlighted in red), production of autoantibodies and subsequent tissue destruction. During autoinflammation, the innate immune cells are hyperactive (highlighted in red) and release pro-inflammatory cytokines, including IL-1β and TNF, which will result in the development of auto-reactive inflammation.

It was not until 1999 that the concept of autoinflammation emerged with the discovery of mutations in TNF receptors by McDermott and colleagues 153. Autoinflammation, just like autoimmunity, leads to the development of self-directed inflammation, but by different mechanisms. Whereas in autoimmune diseases the main player is the adaptive immune system, in autoinflammatory diseases the key role is played by the innate immune system. Autoinflammatory diseases are characterised by hyperactivation of the innate immune system and abnormalities in pro-inflammatory cytokine signalling. One of the molecular mechanisms that has been implicated in the establishment and maintenance of self-tolerance and suppression of the development of autoreactive immune cells is ubiquitination (Fig4).

### E3 ligases in central tolerance

Several E3 ubiquitin ligases have been suggested to be involved in the maintenance of central tolerance 154. Mutation in the AIRE causes the autoimmune polyendocrine syndrome type 1 (APS-1), also called autoimmune polyendocrinophathy-candidiasis-ectodermal dystrophy (APECED), an autosomal recessive disorder characterised by organ-specific autoimmunity that mainly affects endocrine glands 155,156. AIRE is mainly expressed in the nucleus of mTECs, where it regulates the transcription of genes encoding peripheral-tissue antigens (PTAs). This phenomenon, called promiscuous gene expression, is crucial for the establishment of central tolerance 157,158,159. AIRE-deficient mTECs have a specific reduction in the ectopic transcription of genes encoding for PTAs 160. Furthermore, AIRE-deficient mice have decreased expression of a wide array of PTAs and develop circulating autoantibodies, infertility and multi-organ lymphocytic infiltration, mostly in endocrine organs, just like in patients with APECED 161,162,163. AIRE contains two PHD domains that resemble RING finger domains. On the basis of this AIRE has been suggested to function as an E3 ligase 164 but this hypothesis is controversial; on the one hand, missense mutations in PHD1 found in patients with APECED were shown to abolish its E3 ligase activity 164, on the other, the PHD1 domain alone was shown not to have intrinsic E3 ubiquitin ligase activity 165. Further studies will be required to clarify whether AIRE can function as a ubiquitin E3 ligase.

An E3 ligase with a proven role in regulating central self-tolerance is TRAF6. TRAF6-deficient mice display disorganised distribution of mTECs and impairment in their maturation. In addition, expression of AIRE and PTAs were significantly reduced in TRAF6-deficient mice, with the consequent development of an autoimmune phenotype 166. However, it is not clear whether the E3 ligase function of TRAF6 is required for this phenotype. Interestingly, expression of RelB, a member of the NF-κB family, was significantly reduced in thymi of TRAF6-deficient mice 166, and mice with loss of RelB also had thymic abnormalities and multi-organ inflammatory infiltration, suggesting a functional link between TRAF6 and RelB in thymus organogenesis 167. A recent study showed that RANK and CD40 signalling play an important role in the regulation of mTEC development by mediating the activation of TRAF6, NIK, and IKKβ 168.

### E3 ligases in peripheral tolerance

Among the factors known to ensure the maintenance of peripheral tolerance are several E3 ligases including (Cbl)-b and GRAIL). Defects in the expression of these two E3 ligases have been associated with development of autoimmunity, both in human and murine experimental models 169. Cbl-b is a member of the Cbl family, functions as a RING-type E3 ligase 170,171, and is involved in the regulation of T cell tolerance 172,173,174. Cbl-b-deficient mice develop spontaneous autoimmunity characterised by autoantibody production, infiltration of activated T and B lymphocytes into multiple organs, and parenchymal damage 174. Naïve Cbl-b-deficient T cells hyperproliferate and produce IL-2 upon TCR activation, even without CD28 co-stimulation, suggesting a possible role of Cbl-b in the induction of T cell anergy 174. Cbl-b was also shown to be selectively induced during the early phases of T cell unresponsiveness, and Cbl-b-deficient T cells are resistant to anergy induction, both *in vitro* and *in vivo* 175. The molecular mechanism responsible for Cbl-b-driven induction of T cell anergy seems to be Cbl-b-mediated ubiquitination of p85, the regulatory subunit of phosphoinositide 3-kinase (PI3K), with consequent inhibition of p85 recruitment to the cell-surface molecules CD28 and TCRζ 176.

Similar to Cbl-b, the absence of GRAIL from T cells enhances their proliferation and cytokine production after TCR activation in a CD28-independent manner 177. GRAIL is a type I transmembrane protein localised in endosomal compartment that contains a cytosolic enzymatic RING domain and a luminal protease-associated domain 178. It was first identified as a transcript upregulated in anergic CD4^+^ T cells, and it has also been found to be highly expressed in CD4^+^ CD25^+^ T_regs_, where its expression level directly correlates with immunosuppressive activity 178,179. Moreover, its E3 ubiquitin ligase activity is crucial for the induction of T cell anergy 180. *In vivo* studies using GRAIL-deficient mice demonstrated the crucial role of this E3 ligase in maintenance of peripheral tolerance as lack of GRAIL increased susceptibility to autoimmunity 177.

Although several substrates ubiquitinated by GRAIL have been described, including the transmembrane proteins CD40L and the cytosolic Rho GDP dissociation inhibitors (GDIs), the mechanism of GRAIL-mediated anergy induction is not well understood 181,182,183. Furthermore, GRAIL has recently been suggested to regulates T cell tolerance via ubiquitination and consequent degradation of actin-related proteins2/3-5 and coronin 1A, which are actin cytoskeleton-associated proteins 184.

Ich is another E3 ligase up-regulated in anergic T cells 185. It belongs to the Nedd4 family of HECT domain E3 ubiquitin ligases. The crucial role of this protein in the regulation of T cell function has been underscored by the discovery that Itch deficiency in humans correlates with the development of multi-system autoimmune diseases and morphologic and developmental abnormalities. Human Itch deficiency results in a complex phenotype affecting physical growth, craniofacial morphology, muscle development, and immune function 186. Interestingly, Itch-deficient mice have a similar phenotype, characterised by scratching of the skin and immunological disorders, manifested by hyperplasia of lymphoid organs and inflammation in the lung and intestinal tract 187,188. This phenotype is correlated with increased production of IL-4, IL-5, IgG1 and IgE 189. At the molecular level, Itch has been proposed to regulate T cell functions by ubiquitinating several substrates, such as phospholipase Cγ1 and protein kinase Cθ 185, two central players in TCR signalling, as well as JunB, which is required for the transcription of IL-4 189. It has further been shown that Itch mediates the ubiquitination and degradation of Bcl-10, with concomitant inhibition of NF-κB activation in T cells 190.

It will be interesting to determine whether other E3 ligases take part in the maintenance of tolerance, and identify new relevant targets of the known ubiquitin ligases. This will hopefully provide a better understanding of the mechanisms that govern immune tolerance (Sidebar A).

### LUBAC in self-tolerance

As discussed above, LUBAC is involved in several signalling processes, including those triggered by TNF 31,191. Interestingly, mice deficient in SHARPIN, develop chronic proliferative dermatitis (*cpdm*) at 4–6 weeks of age; characterised by inflammatory skin lesion and multi-organ inflammation with a Th2 dominant milieu 191,192. Furthermore, *cpdm* mice also show defective Th1 cytokine production and defects in secondary lymphoid organ development —such as absence of Peyer's patches and marginal zone of the spleen— in which the T and B cell areas are poorly defined 194,195. SHARPIN/TNF-double deficient mice do not develop the *cpdm* skin phenotype and have less liver inflammation, but on the other hand, the immune-development phenotype is unaltered, suggesting that inflammation in *cpdm* mice is TNF-driven, whereas the immune-developmental defect is most likely due to the lack of LUBAC in a different pathway 20.

At first glance, the fact that TNF ablation corrected the inflammatory phenotype that characterises *cpdm* mice may not seem overly surprising, given that inhibition of TNF has been proven to be a highly successful therapeutic approach for the treatment of several chronic inflammatory and autoimmune diseases, including psoriasis, psoriatic arthritis, rheumatoid arthritis and Crohn's disease 196,197,198. However, the benefit of anti-TNF therapy is currently mostly attributed to its interferance with the gene-activatory arm of TNF-signalling. Yet, this arm is attenuated in *cpdm* mice, whilst TNF-induced cell death is increased. Therefore, the rescue from inflammation in *cpdm* mice by co-ablation of TNF had to be due to prevention of aberrant cell death induction, rather than gene activation. This suggests a previously unrecognised aetiology of TNF-induced autoinflammation and/or autoimmunity that relies on supra-physiological levels of cell death induction, rather than aberrantly high gene activation by this cytokine.

Three patients with mutations in the gene that encodes the LUBAC component HOIL-1 were recently identified. These patients presented with a paradoxical clinical phenotype, characterised by the development of an autoinflammatory and immunosuppressed syndrome with pyogenic bacterial diseases and amylopectin-like deposits in muscle, which are known to be a cause of death in the early stages of life 199. To study the basis of autoinflammation and immunosuppression in HOIL-1-deficient patients, Boisson and colleagues analysed the genome-wide transcriptional profiles of whole blood cells and found that erythroid lineage-related transcripts and transcripts encoding pro-inflammatory cytokines were up-regulated in these patients. Additionally, this was correlated with an increase in the plasma concentration of pro-inflammatory cytokines, including IL-6, IL–8, TNF and IL-1β. Whole blood cells from HOIL-1-deficient patients were hyper-responsive to agonists of TLR1/2 and IL-1β stimulation. Indeed, the constitutive hyper-inflammation and hyper-responsiveness to IL-1β could, at least in part, explain the autoinflammatory phenotype of these patients. Furthermore, HOIL-1-deficient patients are also highly susceptible to invasive bacterial infections, which could be due to impairment of NF-κB-dependent responses. This should not come as a surprise, given that patients with inborn errors affecting TLR, IL-1β pathway and NF-κB-mediated immunity —which include NEMO and IκBα—are also prone to pyogenic bacterial infections 200.

### DUBs in self-tolerance

Several DUBs have been shown to be involved in the maintenance of self-tolerance. Mutations in the human A20 locus are associated with autoimmune and inflammatory diseases, including SLE, rheumatoid arthritis and Crohn's disease 201. As mentioned previously, genetic deficiency of A20 leads to multi-organ inflammation, cachexia and neonatal fatality 50. Furthermore, mice lacking A20 in DCs develop anti-DNA antibodies, nephritis and lympho-splenomegaly, all features of SLE 202. Interestingly, three independent single nucleotide polymorphisms (SNPs) in the human A20 region have also been correlated with development of SLE 203. Additionally, loss of A20 in B cells causes an inflammatory syndrome with autoimmune features in old mice characterised by chronic inflammation, high levels of IL-6 and tissue-specific autoantibodies 204.

A20 has been identified to be a susceptibility gene for Crohn's disease by the Wellcome Trust Case Control Consortium in a genome-wide association study for the seven most prevalent common inflammatory diseases conducted on the British population 205. Crohn's disease is a chronic inflammatory bowel disease, most probably occurring due to a deregulation in the immune response to commensal intestinal bacteria. Arsenescu and colleagues analysed the expression of RelA, A20, NF-κB, TNF, IL-8 and pIgR in mucosal biopsies of 69 Crohn's disease patients and 28 non-diseased controls. Based on the expression pattern of these biomarkers, they classified individuals in three groups. Interestingly, patients in group 2 —characterised by low expression of all five biomarkers— had moderate to severe disease and poor responses to immunosuppressive and anti-TNF therapy, suggesting that the use of these biomarkers to classify Crohn's disease patients could help predict disease behaviour and response to therapy 206. Further to this, a recent study identified SNPs in the A20 region of 6q23 as novel coeliac disease associated risk factors 207. An additional independent study, aimed at identifying susceptibility alleles correlating with rheumatoid arthritis, showed an SNP at 6q23 that was also reproducibly associated with rheumatoid arthritis risk 208. In order to define the contribution of A20 to rheumatoid arthritis pathology, Matmati and colleagues generated mice lacking A20 in myeloid cells (A20^myel-KO^) and determined that such mice develop severe features of rheumatoid arthritis-like destructive poly-arthritis, high serum levels of IL-6, TNF, IL-1β and MCP-1 and prolonged NF-κB activation in macrophages, demonstrating the cell-specific function of A20 in causing a rheumatoid arthritis-like pathology 209.

CYLD is another DUB that has been described to play a key role in the regulation of macrophages and T cell activation. CYLD-deficient macrophages show increased NF-κB activity and release high levels of TNF and IL-6 following stimulation by TNF or CD40L 90. Furthermore, CYLD-deficient T cells are hyper-responsive to TCR/CD28 stimulation, leading to spontaneous development of autoimmunity 210. In addition, the adoptive transfer of CYLD-deficient T cells into RAG1-deficient mice was sufficient to induce the inflammatory phenotype, underscoring that the abnormal response of T cells in CYLD-deficient mice was responsible for the occurrence of the inflammatory phenotype.

## Concluding remarks

The ubiquitination of key proteins within receptor signalling complexes and its reversal (their deubiquitination) is essential for eliciting an adequate but confined immune response. Defects in ubiquitination have been implicated in the pathogenesis of a variety of different diseases, including autoimmunity, autoinflammation and immunodeficiency, underlining the importance of its tight regulation and control. In recent years, we have come to appreciate that different variations in the theme of ubiquitination and deubiquitination are responsible for this. We are, however, far from fully understanding these processes, both molecularly and functionally. We predict that further elucidation of the precise role of individual ubiquitination and deubiquination events in regulating immune signalling will be necessary to understand how and why aberrant ubiquitin signalling outputs result in a disturbed host response. Deeper insights into these processes probably also reveal novel therapeutic options for the treatment of diseases and pathological conditions in which the immune system does not act to the full extent of its capacity, or within its physiologically defined limits.

**“Ubiquitylation: mechanism and functions” Review series**

**Previous issues of EMBO reports include:**

- Building and remodeling Cullin-RING E3 ubiquitin ligases, by Wade Harper *et al*

**Other reviews in this series, which will be published in consecutive issues of EMBO reports, will cover:**

- RBR E3 ligases at work, by Judith Smit and Titia Sixma
- Dynamic survey of mitochondria by ubiquitin, by Mafalda Escobar-Henriques and Thomas Langer
- Ubiquitylation in stem cells, by Ioannis Aifantis *et al*
- Understanding ubiquitylation one structure at a time, by Ronald Hay *et al*

## Footnotes

* Correction added after publication 27 December 2013: in the definition of IPS in the Glossary, IFNα was corrected to IFN-β

## Abbreviations

M1: Linear linkages
ABIN1: A20 binding inhibitor of NF-κB 1
AIRE: Autoimmune regulator protein
AMPK: AMP-activated protein kinase
CARDIF: CARD adaptor inducing IFN-β
Cbl-B: Casitas B-lineage lymphoma
cFLIP: Cellular FLICE/caspase-8 inhibitory protein
cIAP: Cellular inhibitor of apoptosis protein
DUBs: Deubiquitinating enzymes
FADD: Fas-associated protein with death domain
GRAIL: Gene related to anergy in lymphocytes
HECT: Homology to E6AP C-terminus
HHARI: human homologue of Drosophila ariadne
HOIL-1: Heme-oxidised iron-responsive element-binding protein 2 ubiquitin ligase-1
HOIP: HOIL-1-interacting protein
IKK: Inhibitor of κB kinase
IPS: IFN-β promoter stimulator*
IRAK: IL1-R-associated kinase
LGP2: Laboratory of genetics and physiology gene 2
LUBAC: Linear ubiquitin chain assembly complex
MAVS: Mitochondrial antiviral signalling protein
MDA5: Melanoma differentiation-associated gene 5
MyD88: Myeloid differentiation primary response gene 88
Nedd4: Neuronal precursor cell-expressed developmentally downregulated 4
NEMO: NF-κB essential modulator
NIK: NF-κB-inducing kinase
NOD: Nucleotide oligomerization domain
NZF: Npl4 zinc finger
OTU: Ovarian tumour proteases
OTUB1: OTU domain-containing ubiquitin aldehyde-binding protein 1
OTULIN: OTU DUB with linear linkage specificity
PHD: Plant homeodomain
pIgR: Polymeric immunoglobulin receptor
PRR: Pattern recognition receptor
RANK: Receptor activator of NF-κB
RIG-I: Retinoic acid-inducible gene
RBR: RING-between-RING
RING: Really Interesting New Gene
RIP: Receptor interacting protein
SHARPIN: SH3 and multiple ankyrin repeat domains protein (SHANK)-associated RBCK1 homology (RH)-domain-interacting protein
TAB: TAK1-binding protein
TAK: Transforming growth factor-β activated kinase 1
TAXBP1: Human T-cell leukemia virus type I (Tax1) binding protein 1
TBK1: Tank-binding kinase 1
TIEG1: Transforming growth factor-β-inducible early growth response protein 1
TNF: Tumour necrosis factor
TNFR1: (TNF receptor 1)
TNF-RSC I: (TNF-receptor signalling complex I)
TRABID: TRAF-binding-domain-containing protein
TRADD: TNFR1-associated death-domain (DD)
TRAF: TNF receptor-associated factor
TRIF: TIR-domain-containing adaptor protein inducing interferon-β-mediated transcription-factor
TRIM25: Tripartite motif containing protein 25
UBAN: Ubiquitin binding in ABIN and NEMO
VISA: Virus-induced signalling adaptor
XIAP: X-linked inhibitor of apoptosis protein
